# Cost-Effectiveness of Robot-Assisted Radical Cystectomy vs Open Radical Cystectomy for Patients With Bladder Cancer

**DOI:** 10.1001/jamanetworkopen.2023.17255

**Published:** 2023-06-30

**Authors:** Simon Dixon, Harry Hill, Laura Flight, Pramit Khetrapal, Gareth Ambler, Norman R. Williams, Chris Brew-Graves, John D. Kelly, James W. F. Catto

**Affiliations:** 1 School of Health and Related Research, University of Sheffield, Sheffield, England; 2 PRICELESS SA (Priority Cost Effective Lessons for System Strengthening South Africa), School of Public Health, University of the Witwatersrand, Johannesburg, South Africa; 3 National Institute for Health Care Excellence, Manchester, England; 4 Division of Surgery & Interventional Science, University College London, London, England; 5 Department of Statistical Science, University College London, London, England; 6 Surgical & Interventional Trials Unit, Division of Surgery & Interventional Science, University College London, London, England; 7 Department of Oncology and Metabolism, University of Sheffield, Sheffield, England; 8 Department of Urology, Sheffield Teaching Hospitals NHS (National Health Service) Foundation Trust, Sheffield, England

## Abstract

**Importance:**

The value to payers of robot-assisted radical cystectomy with intracorporeal urinary diversion (iRARC) when compared with open radical cystectomy (ORC) for patients with bladder cancer is unclear.

**Objectives:**

To compare the cost-effectiveness of iRARC with that of ORC.

**Design, Setting, and Participants:**

This economic evaluation used individual patient data from a randomized clinical trial at 9 surgical centers in the United Kingdom. Patients with nonmetastatic bladder cancer were recruited from March 20, 2017, to January 29, 2020. The analysis used a health service perspective and a 90-day time horizon, with supplementary analyses exploring patient benefits up to 1 year. Deterministic and probabilistic sensitivity analyses were undertaken. Data were analyzed from January 13, 2022, to March 10, 2023.

**Interventions:**

Patients were randomized to receive either iRARC (n = 169) or ORC (n = 169).

**Main Outcomes and Measures:**

Costs of surgery were calculated using surgery timings and equipment costs, with other hospital data based on counts of activity. Quality-adjusted life-years were calculated from European Quality of Life 5-Dimension 5-Level instrument responses. Prespecified subgroup analyses were undertaken based on patient characteristics and type of diversion.

**Results:**

A total of 305 patients with available outcome data were included in the analysis, with a mean (SD) age of 68.3 (8.1) years, and of whom 241 (79.0%) were men. Robot-assisted radical cystectomy was associated with statistically significant reductions in admissions to intensive therapy (6.35% [95% CI, 0.42%-12.28%]), and readmissions to hospital (14.56% [95% CI, 5.00%-24.11%]), but increases in theater time (31.35 [95% CI, 13.67-49.02] minutes). The additional cost of iRARC per patient was £1124 (95% CI, −£576 to £2824 [US $1622 (95% CI, −$831 to $4075)]) with an associated gain in quality-adjusted life-years of 0.01124 (95% CI, 0.00391-0.01857). The incremental cost-effectiveness ratio was £100 008 (US $144 312) per quality-adjusted life-year gained. Robot-assisted radical cystectomy had a much higher probability of being cost-effective for subgroups defined by age, tumor stage, and performance status.

**Conclusions and Relevance:**

In this economic evaluation of surgery for patients with bladder cancer, iRARC reduced short-term morbidity and some associated costs. While the resulting cost-effectiveness ratio was in excess of thresholds used by many publicly funded health systems, patient subgroups were identified for which iRARC had a high probability of being cost-effective.

**Trial Registration:**

ClinicalTrials.gov Identifier: NCT03049410

## Key Points

**Question:**

Is robot-assisted radical cystectomy with intracorporeal urinary diversion cost-effective compared with open radical cystectomy for patients with bladder cancer?

**Findings:**

In this economic evaluation of a randomized clinical trial including 305 patients, robot-assisted surgery was associated with reductions in admissions to intensive care and readmissions to hospital but increases in theater time. Robot-assisted cystectomy has an incremental cost-effectiveness ratio of £100 008 (US $144 312) per quality-adjusted life-year gained, but subgroups defined by age, tumor stage, and performance status have much higher probabilities of being cost-effective.

**Meaning:**

These findings suggest that payers need to consider the role of patient subgroups when assessing coverage decisions for this indication.

## Teaser

This economic evaluation of a randomized clinical trial compares the cost-effectiveness of intracorporeal robot-assisted radical cystectomy with that of open radical cystectomy for patients with bladder cancer, including the consideration of prespecified patient subgroups.

## Introduction

Each year more than 550 000 new cases of bladder cancer are diagnosed worldwide.^1^ Around one-third of bladder cancers require radical treatment, including radical cystectomy with pelvic lymphadenectomy.^2^ An estimated 33 429 radical cystectomy operations were performed in the US from 2008 to 2011,^3^ with most patients developing 1 or more complications and 20% to 30% readmitted post discharge.^4,5^ While reductions in morbidity from radical cystectomy have been achieved through robot-assisted radical cystectomy (RARC),^6,7^ the cost-effectiveness of these approaches is unclear.

A systematic review of economic studies of RARC and open radical cystectomy (ORC)^8^ highlighted that the costs of the robot-assisted procedure are likely to be higher than open comparators, despite savings from reductions in complications. However, changes associated with patient throughput and shorter surgery times were highlighted as important considerations, as well as the need for prospectively collected quality of life information that could be used to generate quality-adjusted life-years (QALYs). A prospectively designed cost-effectiveness analysis that was integrated into a randomized comparison of total intracorporeal RARC (iRARC) and ORC provides this information. The objective of this study was to compare the cost-effectiveness of iRARC with that of ORC for patients with bladder cancer, including the consideration of prespecified patient subgroups.

## Methods

### Patients and the iROC Study

The cost-effectiveness analysis was integrated into the iROC (Intracorporeal Robot-Assisted Radical Cystectomy vs Open Radical Cystectomy) study, which was a multicenter, unblinded, randomized clinical trial that recruited patients from 9 centers in the United Kingdom from March 20, 2017, to January 29, 2020.^6^ The primary objective of the trial was to investigate the effects of the different forms of surgery on patient recovery. Patients were eligible to be recruited to the trial if they were adults with nonmetastatic urothelial, squamous, adenocarcinoma, or variant bladder cancer. Of 338 patients randomized (169 in each group), 317 underwent radical cystectomy, with those in the robot-assisted group spending a mean of 2.2 (95% CI, 0.50-3.85) days longer alive or out of hospital. Statistically significant differences in health-related quality of life (HRQOL) and disability were also identified at 5 weeks using the European Quality of Life 5-Dimension 5-Level instrument (EQ-5D-5L)^9^ and the World Health Organization Disability Assessment Schedule, version 2.0.^10^ The trial received ethical approval from the Newcastle and North Tyneside Research Ethics Committee, and all patients gave written informed consent (for details, see Catto et al^6^). Reporting aligned with Consolidated Health Economic Evaluation Reporting Standards (CHEERS) and is detailed in eMethods in Supplement 1.

### Cost-Effectiveness Analysis

The cost-effectiveness analysis used QALYs based on patient EQ-5D-5L responses and was undertaken from the perspective of the United Kingdom National Health Service.^11^ When analyzed as described, EQ-5D-5L scores range from 1.00 (perfect health) through 0 (a health state equivalent to death) to −0.594 (the worst possible health classified by the instrument). The primary analysis used a time horizon of 90 days post surgery so that it aligned with the trial evidence, and secondary analyses were based on projections of patient recovery to 180, 270, and 360 days. The general approach was consistent with methodological guidelines^12^ outlined in the trial protocol^13^ and prespecified in a health economics analysis plan (eMethods in Supplement 1). Costs have been converted to US dollars using the purchasing power parity for 2021.^14^

### Resource Use

The principal differences in resource use were expected to be related to theater equipment, staff mix in the theater, length of theater time, and length of ward stay. Further differences were considered in terms of intensive care, high-dependency care, units of blood transfused, family physician attendances, emergency department attendances, and readmissions. Data were available via case report forms from the iROC trial and relate to the period from admission to the theater suite. Length of stay was calculated as the number of separate days on which a patient was present in hospital.

### Unit Costs

All unit costs are based on fully absorbed accounting principles, or market prices if available; no hospital charges were used. Robot costs were based on the purchase and maintenance price for a surgical robot (Da Vinci X; Intuitive), a simulator, instruments, and staff time for training. Nonrecurrent costs were annuitized using a discount rate of 3.5% in advance over 10 years and zero reuse value.^11^ Capital costs were allocated across 206 patients per annum, based on a study by Lam et al,^15^ and surgeon training costs were allocated across 40 patients per annum. The resultant cost for iRARC was £2638 (US $3807) per patient (eTable 1 in Supplement 1). Equipment costs for ORC were taken from a recent United Kingdom–based study that identified and priced each individual component of theater equipment used, which produced a cost of £1514 (US $2185) per patient.^16^

Cost per theater minute and cost per ward day were calculated in consultation with business managers at one of the larger recruiting sites. Theater costs were based on staffing, equipment, and consumables in urology theaters. Both theater and ward costs were then adjusted, pro rata, to match national average costs (eTable 2 in Supplement 1). All other unit costs were taken from publicly available sources (eTable 2 in Supplement 1). All costs were at 2020-2021 price levels, with adjustment to that level if required, using the National Health Service Cost Inflation Index.^17^

### Outcomes

We calculated QALYs using linear interpolation of EQ-5D-3L United Kingdom tariff values. Tariff values were calculated using the van Hout crosswalk tariff.^18^ The EQ-5D-5L was completed at baseline and 5 weeks and 90 days post surgery. However, this was not considered to be adequate for incorporating potential short-term differences in HRQOL, and so EQ-5D-5L values at 5 days post surgery were imputed using quantified activity levels recorded at that time. Specifically, using the aldvmm package in R, version 4.2.2 (R Project for Statistical Computing), an adjusted limited dependent variable mixture regression model was fitted between the week 5 EQ-5D-5L and week 4 activity data (mean across days 4, 5, and 6), together with appropriate covariates. The results were then used to estimate the day 5 EQ-5D-3L score using the mean activity data across the 3 days.

### Statistical Analysis

Data were analyzed from January 13, 2022, to March 10, 2023. Mean resource use was estimated and compared using unpaired, 2-tailed *t* tests with unequal variances for continuous variables, negative binomial regression for count data, and χ^2^ tests for event rates. A 5% level of statistical significance was used with 2-sided hypothesis tests (*P* < .05). Costs and QALYs were compared using seemingly unrelated regression models to account for correlation between costs and QALYs.^19^ For cost, the sole independent variable was treatment group, while for QALYs, treatment group, age, sex, and baseline EQ-5D-5L utility score were used as independent variables. Subgroup analyses incorporated an interaction term into the same regression specification. The analysis was based on patients for whom the primary outcome of the clinical trial was available (n = 305).

Missing data for EQ-5D-5L utility score (at baseline, 5 days, 5 weeks, and 12 weeks) were based on 20 imputed data sets. The imputed data sets were established in chain regressions with covariates of age, sex, and group. All analyses, unless otherwise stated, were undertaken in Stata, version 17 (StataCorp LLC).

Incremental cost-effectiveness ratios (ICERs) were calculated using the coefficients of the treatment variables of the seemingly unrelated regressions. Five thousand mean values for incremental costs and QALYs were bootstrapped and plotted on the cost-effectiveness plane to give a cost-effectiveness acceptability curve. The main conclusions of the analysis are based on a United Kingdom funding threshold of £20 000 (US $28 8960) per QALY gained.^11^

Preplanned subgroup analyses included the following: chemotherapy vs no chemotherapy, stage T2 or less vs T3 or greater, age younger than 70 years vs 70 years or older, performance status 0 vs 1 or greater on the ECOG Performance Status Scale (score range, 0 [fully active] to 5 [dead]), male vs female sex, and type of diversion, consisting of ileal conduit vs neobladder or other reconstruction. A further post hoc analysis was undertaken to assess where body mass index (BMI; calculated as weight in kilograms divided by height in meters squared) may be a potential effect modifier.

Sources of methodological and cost uncertainty were identified and explored through deterministic sensitivity analyses. Sources relating to methodological uncertainty included:

- complete case analysis (cases with complete EQ-5D-5L data at baseline and 5 weeks and 90 days post surgery);
- omission of the day 5 utility imputation;
- use of last observation carried forward for missing data;
- scoring the EQ-5D-5L using an alternative algorithm^20^; and
- extrapolation of day 90 results assuming convergence of mean utilities at 180, 270, and 360 days post surgery.

Sources relating to cost uncertainty are detailed in eTable 3 in Supplement 1, but briefly included:

- lower theater cost per minute for iRARC;
- lower ORC equipment costs;
- lower cost of a day on a ward;
- alternative life span and throughput of the surgical robot; and
- alternative hospital costs.

## Results

Data were available for 305 patients, all of whom were included in the analysis. Race and ethnicity data were not collected as there is no robust evidence that race or ethnicity is related to prognosis. Mean (SD) age was 68.3 (8.1) years; 241 (79.0%) were men and 64 (21.0%) were women.

### Costs of ORC and iRARC

**Figure 1.**
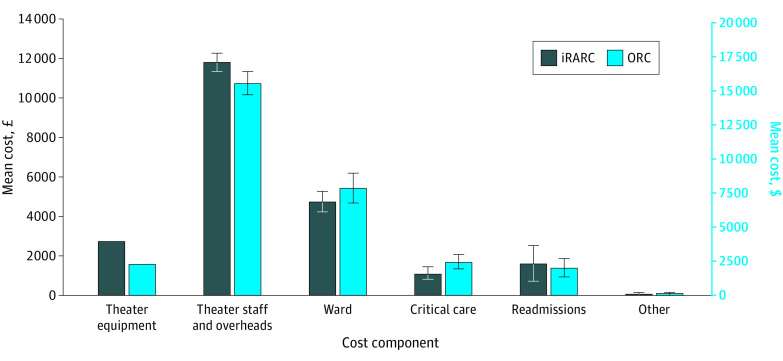
Breakdown of Total Costs by Treatment and Cost Component Treatment groups include intracorporeal robot-assisted radical cystectomy (iRARC) and open radical cystectomy (ORC). Error bars indicate 95% CIs.

As shown in Table 1, theater time was 31.35 minutes longer for iRARC than for ORC (95% CI, 13.67-49.02 minutes; *P* < .001). Conversely, iRARC resulted in 6.35% fewer admissions to an intensive therapy unit (95% CI, 0.42%-12.28%; *P* = .04) and 14.56% fewer postdischarge readmissions to the hospital (95% CI, 5.00%-24.11%; *P* = .003). Ward days and admissions to high-dependency care are lower for iRARC than ORC, although comparisons were not statistically significant. The staff mix of surgeons also differed between trial groups; 39 of 156 robotic procedures (25.00%) were undertaken by a consultant alone (with nursing assistance), compared with 16 of 148 (10.81%) for ORC (χ^2^ test; *P* = .02) (eTable 4 in Supplement 1). When combined with unit costs and summed to produce a total cost per patient, these differences partially offset the additional cost of the surgical robot (Figure 1) to produce an additional cost of iRARC of £1124 (95% CI, −£576 to £2824 [US $1622 (−$831 to $4075)]; *P* = .20) (Table 2).

**Table 1.** The Difference in Resource Use for the ORC and iRARC Interventions

Resource	Intervention group		Increment^a^	*P* value (test)
	ORC (n = 148)	iRARC (n = 157)		
Theater minutes, mean (SD)	267.53 (94.11)	298.87 (72.84)	−31.35	<.001 (*t* test)^b^
Ward days, mean (SD)	10.13 (8.77)	8.84 (6.29)	1.29	.07 (NBR)
Units of blood, mean (SD)	0.32 (0.94)	0.26 (1.72)	0.06	.62 (NBR)
Admitted to ITU, No. (%)	16 (10.81)	7 (4.46)	6.35	.04 (χ^2^ test)
Admitted to HDU, No. (%)	52 (35.14)	41 (26.11)	9.03	.09 (χ^2^ test)
Readmission, No. (%)	47 (31.76)	27 (17.20)	14.56	.003 (χ^2^ test)
Attendance at emergency department, No. (%)	43 (29.05)	37 (23.57)	5.48	.28 (χ^2^ test)
Attendance with family physician, No. (%)	65 (43.92)	77 (49.04)	−5.12	.37 (χ^2^ test)

Abbreviations: HDU, high-dependency unit; iRARC, intracorporeal robot-assisted radical cystectomy; ITU, intensive therapy unit; NBR, negative binomial regression; ORC, open radical cystectomy.

^a^ Relates to the percentages in the 2 preceding columns.

^b^ Indicates unpaired 2-tailed *t* test with unequal variance.

**Table 2.** Sensitivity Analysis of Incremental Costs, QALYs, and Cost-Effectiveness of iRARC vs ORC

Analysis	Incremental cost				ICER^a^	Cost-effective probability, %^b^	No. of patients
	QALY (95% CI)	*P* value	Cost (95% CI)	*P* value			
Primary analysis	0.01124 (0.00391 to 0.01857)	.003	£1124 (−£576 to £2824 [US $1622 (−$831 to $4075)])	.20	£100 008 (US $144 312)	16.3	305
Sensitivity analyses 1-5 (methodological uncertainty)							
Complete case analysis	0.01153 (0.00226 to 0.02081)	.02	−£88 (−£1800 to £1624 [US −$127 (−$2597 to $2343)])	.92	iRARC dominant	63.3	196
Omitted day 5 imputation	0.01015 (0.00142 to 0.02066)	.005	£1124 (−£576 to £2824 [US $1622 (−$831 to $4075)])	.20	£110 738 (US $159 795)	14.9	305
Last observation carried forward imputation	0.01204 (0.00313 to 0.02094)	.008	£626 (−£978 to £2231 [US $903 (−$1411 to $3219)])	.44	£52 020 (US $75 065)	32.0	240
Application of an alternative EQ-5D-5L tariff	0.01509 (0.00850 to 0.02169)	.001	£1124 (−£576 to £2824 [US $1622 (−$831 to $4075)])	.20	£74 459 (US $107 444)	18.1	305
90 d of extrapolation	0.01374 (0.00641 to 0.02107)	.001	£1124 (−£576 to £2824 [US $1622 (−$831 to $4075)])	.20	£81 820 (US $118 066)	17.3	305
180 d of extrapolation	0.01624 (0.00890 to 0.02357)	.001	£1124 (−£576 to £2824 [US $1622 (−$831 to $4075)])	.20	£69 219 (US $99 883)	18.7	305
270 d of extrapolation	0.01874 (0.01141 to 0.02607)	.001	£1124 (−£576 to £2824 [US $1622 (−$831 to $4075)])	.20	£59 968 (US $86 534)	19.9	305
Sensitivity analyses 6-9 (cost uncertainty)							
Theater consumable cost for ORC group at 75% value	0.01124 (0.00391-0.01857)	.003	£1502 (−£102 to £3101 [US $2167 (−$147 to $4475)])	.08	£133 630 (US $192 828)	7.7	305
Theater consumable cost for ORC group at 50% value	0.01124 (0.00391 to 0.01857)	.003	£1881 (£127 to £3527 [US $2714 ($183 to $5089)])	.03	£167 349 (US $241 485)	3.1	305
Cost of ward day at lower value to reflect marginal cost of saved days	0.01125 (0.00392 to 0.01858)	.003	£1361 (−£205 to £2926 [US $1964 (−$296 to $4222)])	.09	£120 957 (US $174 541)	7.8	305
Decreased robotic surgery times by 5%	0.01125 (0.00391 to 0.01856)	.003	£551 (−£1139 to £2242 [US $795 (−$1644 to $3235)])	.52	£49 039 (US $70 763)	36.1	305
Decreased robotic surgery times by 10%	0.01125 (0.00391 to 0.01856)	.003	−£21 (−£1702 to £1660 [US −$30 (−$2456 to $2395)])	.98	iRARC dominant	61.9	305
Robotic surgery: longer longevity of use with higher throughput	0.01124 (0.00391 to 0.01857)	.003	£796 (−£904 to £2496 [US $1149 (−$1304 to $3602)])	.36	£70 819 (US $102 192)	26.1	305
Robotic surgery: shorter longevity of use with lower throughput	0.01124 (0.00391 to 0.01857)	.003	£1826 (£127 to £3527 [US $2635 ($183 to $5089)])	.04	£162 505 (US $234 495)	3.3	305
Cost per minute of theater and ward cost at lower quartile national costs	0.01125 (0.00392 to 0.01858)	.003	£965 (−£408 to £2338 [US $1392 (−$589 to $3374)])	.17	£85 807 (US $123 820)	14.1	305
Cost per minute of theater and ward cost at upper quartile national costs	0.01123 (0.00390 to 0.01856)	.003	£1207 (−£688 to £3103 [US $1742 (−$993 to $4478)])	.21	£107 480 (US $155 094)	16.8	305

Abbreviations: EQ-5D-5L, European Quality of Life 5-Dimension 5-Level instrument; ICER, incremental cost-effectiveness ratio; iRARC, intracorporeal robot-assisted radical cystectomy; ORC, open radical cystectomy; QALY, quality-adjusted life-year.

^a^ iRARC dominant indicates that iRARC is more effective and less costly, and in such cases, an ICER is not generally reported due to problems associated with the interpretation of negative ratios.

^b^ Indicates the probability that iRARC is cost-effective at £20 000 per QALY gained.

### QALYs and Cost-Effectiveness

**Figure 2.**
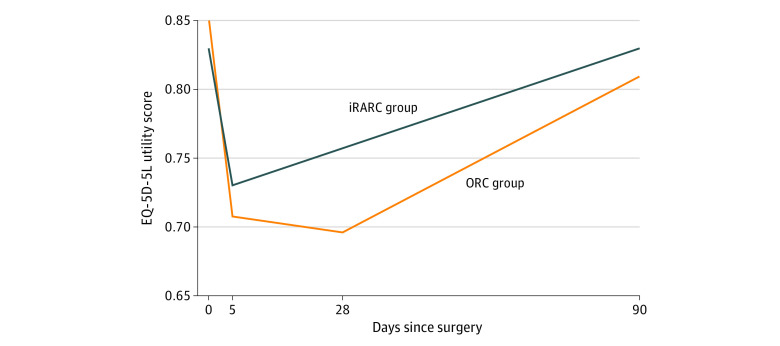
European Quality of Life 5-Dimension 5-Level Instrument (EQ-5D-5L) Responses to 90 Days, Including Day 5 Imputation When analyzed as described, EQ-5D-5L scores range from 1.00 (perfect health) through 0 (a health state equivalent to death) to −0.594 (the worst possible health classified by the instrument). iRARC indicates robot-assisted radical cystectomy with intracorporeal urinary diversion; ORC, open radical cystectomy.

The profile of HRQOL as measured by EQ-5D-5L is shown in Figure 2. The QALYs associated with these profiles, calculated as the areas under the curves, produce an incremental benefit associated with iRARC of 0.01124 QALYs (95% CI, 0.00391-0.01857 QALYs; *P* = .003). The ICER is £100 008 (US $144 312) per QALY gained (Table 2), with a 16.3% chance that iRARC is cost-effective at £20 000 (US $28 860) per QALY gained. The cost-effectiveness plane and associated cost-effectiveness acceptability curves are shown in eFigures 1 and 2 in Supplement 1, respectively.

### Sensitivity Analysis

Sensitivity analyses relating to methodological uncertainties (Table 2) revealed changes associated with the ICER of alternative approaches to imputation. The use of a complete case analysis yielded the greatest changes, suggesting that iRARC reduces cost and improves health (dominant), increasing the probability that iRARC is cost-effective to 63.3%. Use of the last observation carried forward as an imputation method had a less dramatic effect, showing a 32.0% chance of iRARC being cost-effective. The extrapolation of health benefits to 1 year after surgery (90 days of patient follow-up plus 270 days of extrapolation) was associated with a 19.9% chance of being cost-effective (Table 2).

Changes in unit costs reflected iRARC surgery times being shorter than those observed in iROC, with the greatest changes to the ICER; a 10% reduction showed that iRARC was dominant and had a 61.9% chance of being cost-effective at £20 000 (US $28 860) per QALY gained. The estimated changes in robot life expectancy and throughput yielded smaller changes to the ICER. Lower ward costs that may better reflect marginal cost savings yielded only small changes to the ICER. The results appear generalizable to higher- and lower-cost hospitals, with the use of United Kingdom upper and lower quartile costs yielding a small change to the ICERs. These results are shown in Table 2.

### Subgroup Analysis

The subgroup analyses revealed large changes to cost-effectiveness when different age groups, tumor stages, and performance status were evaluated (Table 3). The probabilities of iRARC being cost-effective at £20 000 (US $28 860) per QALY gained were 82.2% among patients 70 years or older, 77.6% among those with large tumors, and 84.7% among those with a worse performance status. There were moderate improvements in cost-effectiveness among patients undergoing an ileal conduit diversion, with an ICER of £58 101 (US $83 840) per QALY gained and probability of being cost-effective of 32.9%, and those with a BMI of 25 or above, with an ICER of £66 656 (US $96 185) per QALY gained and probability of being cost-effective of 34.1%.

**Table 3.** Subgroup Analyses of Incremental Costs, QALYs, and Cost-Effectiveness of iRARC vs ORC

Subgroups	Incremental				ICER^a^	Cost-effective probability, %^b^	No. of patients
	QALY (95% CI)	*P* value	Cost (95% CI)	*P* value			
Chemotherapy							
Yes	0.01459 (0.00228 to 0.02690)	.02	£1712 (−£1189 to £4612 [US $2470 (−$1716 to $6655)])	.25	£117 353 (US $169 341)	15.4	104
No	0.00952 (0.00051 to 0.01853)	.04	£818 (−£1276 to £2912 [US $1180 (−$1841 to $4202)])	.49	£85 940 (US $124 012)	30.0	201
Tumor stage							
≥T3	0.00516 (−0.00949 to 0.01981)	.50	−£1303 (−£4621 to £2015 [US −$1880 (−$6655 to $2908)])	.45	iRARC dominant	77.6	75
≤T2	0.01295 (0.00395 to 0.02197)	.005	£1970 (−£54 to £3994 [US $2843 (−$78 to $5763)])	.06	£152 069 (US $219 436)	4.8	205
Age							
≥70 y	0.01327 (0.00317 to 0.02337)	.01	−£855 (−£1468 to £3178 [US −$1234 (−$2118 to $4586)])	.48	iRARC dominant	82.2	154
<70 y	0.01042 (0.00064 to 0.02021)	.04	£3340 (£956 to £5723 [US $4820 ($1380 to $8258)])	.006	£320 377 (US $462 304)	0.7	151
Performance status							
≥1	0.01892 (0.00180 to 0.03604)	.03	−£1807 (−£2425 to £6039 [US −$2608 (−$3499 to $8714)])	.41	iRARC dominant	84.7	51
0	0.01071 (0.00236 to 0.01906)	.01	£1899 (−£176 to £3973 [US $2740 (−$254 to $5733)])	.07	£177 343 (US $255 906)	6.2	221
Sex							
Women	0.00436 (−0.01100 to 0.01972)	.59	£794 (−£2822 to £4410 [US $1146 (−$4072 to $6364)])	.68	£182 220 (US $262 944)	32.7	64
Men	0.01305 (0.00110 to 0.02500)	.002	£1249 (−£655 to £3153 [US $1802 (−$945 to $4550)])	.20	£95 711 (US $138 111)	16.9	241
Type of diversion							
Ileal conduit	0.01062 (0.00278 to 0.01847)	.008	£635 (−£1090 to £2360 [US $916 (−$1573 to $3405)])	.48	£59 791 (US $86 278)	32.9	266
Neobladder or other	0.01964 (−0.00170 to 0.04098)	.07	£5177 (£337 to £10 018 [US $7470 ($486 to $14 456)])	.04	£263 629 (US $380 417)	2.2	35
Body mass index^c^							
Overweight/ or obese (≥25)	0.01011 (0.00158 to 0.01863)	.02	£659 (−£1299 to £2617 [US $951 (−$1874 to $3776)])	.52	£65 232 (US $94 130)	34.1	215
Underweight or normal weight (<25)	0.01411 (0.00074 to 0.02748)	.04	£2204 (−£901 to £5309 [US $3180 (−$1300 to $7661)])	.16	£156 222 (US $225 429)	8.0	90

Abbreviations: ICER, incremental cost-effectiveness ratio; iRARC, intracorporeal robot-assisted radical cystectomy; ORC, open radical cystectomy; QALY, quality-adjusted life-year.

^a^ iRARC dominant means that iRARC is more effective and less costly, and in such cases, an ICER is not generally reported due to problems associated with the interpretation of negative ratios.

^b^ Indicates the probability that iRARC is cost-effective at £20 000 per QALY gained.

^c^ Calculated as weight in kilograms divided by height in meters squared.

An exploratory post hoc analysis was undertaken to examine individual cost components and how they differed between patient subgroups (eTable 5 in Supplement 1). Our findings suggest that the improved cost-effectiveness of iRARC for more elderly patients was associated with larger reductions in length of stay and readmissions. Reductions in length of stay were also present for patients with larger tumors, while for performance status, there appeared to be no clear association.

## Discussion

Our primary economic analysis suggests that iRARC has greater costs per procedure than ORC. There are clear differences in resources used by each surgical approach, with the higher equipment costs of iRARC being partly offset by savings in other ward and critical care costs. This higher cost per patient (£1124 [US $1622]; *P* = .20) was associated with greater health benefits (0.01124 QALYs; *P* = .003); however, the resultant ICER of £100 008 (US $144 312) was above normal funding thresholds in the United Kingdom.

Our sensitivity analysis highlighted several practical issues. First, reducing robotic surgical times has a large effect on cost-effectiveness. Second, more general changes in the level of health service costs (excluding equipment prices) had relatively small effects due to the counteracting effects of iRARC producing higher theater costs but lower ward costs. However, it should be recognized that these conclusions are based on changes in one cost component at a time. For example, if it is possible to reduce the additional cost of iRARC by £899 (US $1297) per patient by a combination of changes to working practices and/or price, then iRARC becomes cost-effective at a threshold of £20 000 (US $28 860) per QALY gained.

Subgroup analyses revealed large changes in cost-effectiveness; iRARC was cost-effective in patients 70 years or older, with tumor stages of T3 or greater, or with a performance status of 1 or above. Our exploratory analysis of these findings suggests that some of these differences are due to younger patients being able to tolerate open surgery better, and so the length of hospital stay and readmission benefits of iRARC are much greater in the older patient group. However, given the post hoc nature of these analyses and the correlations between subgroup membership, we do not believe that it is appropriate to tease apart these differences further.

Nonetheless, the subgroup analysis suggests that even if payers consider iRARC not to be cost-effective for all patients within the iROC study, subgroups can be identified for whom iRARC is cost-effective. Other subgroups relating to type of diversion and BMI may also be cost-effective, depending on the funding thresholds adopted by various countries or health plans.

### Strengths and Limitations

The biggest strength of this study is that it was integrated into a high-quality randomized clinical trial with patient-level data for all major cost components and HRQOL. As such, it overcomes the weaknesses identified in a previously published systematic review of robot-assisted radical cystectomy for patients with bladder cancer.^8^ In addition, the subgroup analyses yielded information for payers who may wish to limit coverage for economic or budget impact reasons.

The incorporation of patient quality of life into our analysis using QALYs is in line with the recommendation of prominent bodies in Canada, the United Kingdom, and the US.^11,21,22,23^ The quality adjustment was undertaken using the EQ-5D-5L, which is used extensively as a patient-reported outcome measure in cancer trials.^24,25,26,27^

The economic evaluation was also undertaken with direct and indirect stakeholder involvement to ensure its relevance. It was designed with the assistance of the lead clinician of the underlying iROC study, while the final design and conduct of the iROC study was overseen by a steering committee that included patient representatives.^13^

The main limitation relating to the clinical evidence is the use of the 90-day follow-up, which is expected to systematically underestimate the HRQOL benefits of iRARC. However, sensitivity analysis explored this, and with extrapolation to what was considered to be the largest plausible length of morbidity benefit (360 days), the ICER remained high. The main limitation for the costs was the lack of study data on the use and cost of instruments for ORC, which meant relying on a previously published figure. While this figure was the most relevant figure available, other available estimates for ORC and related surgical procedures are much lower. This was explored in the sensitivity analysis.

The generalizability of costs and cost-effectiveness needs to be considered by funders. While our sensitivity analysis showed that variability in costs has a limited effect, this is based on United Kingdom cost structures and surgical practices. As such, the extent to which these economic results can be generalized beyond the United Kingdom is unknown. Similarly, the funding threshold presented may not be relevant to other countries (even if translated to local currency units). Consequently, local information needs to be taken into account when our results are used to inform policy in other countries. This is perhaps most pertinent to length of stay, as hospitals and/or countries with short lengths of stay may be less likely to be able to deliver the absolute reductions seen here, which will lead to reduced cost-effectiveness.

There were 5 deviations from the health economics analysis plan. Three of these relate to the adoption of alternative methods: the choice of hospitals used to estimate costs, the method of extrapolation beyond the trial follow-up, and the source of equipment costs relating to ORC. All changes were explored with sensitivity analysis and were found to alter the results only minimally. The other 2 changes relate to the subgroup analysis of BMI and the exploratory analysis relating to the subgroups analysis of age, tumor size, and performance status. These are clearly reported as post hoc analyses with findings interpreted accordingly.

## Conclusions

The findings of this economic evaluation suggest that iRARC was more effective in reducing short-term morbidity compared with ORC for patients with bladder cancer. This was mirrored by reductions in inpatient stay, admissions to an intensive therapy unit, and readmissions. However, these cost offsets were smaller than the cost increases associated with theater time and equipment. The resulting ICER was in excess of thresholds set by most publicly funded health systems and schemes; however, patient subgroups were identified for which iRARC had a probability of being cost-effective of more than 75%. Future research should examine patient subgroups and service settings where iRARC is most cost-effective, including an assessment of recovery using patient-reported outcome measures.

## Notes

Supplement 1. **eMethods.** Health Economics Analysis Plan **eTable 1.** Unit Cost of Robot per Patient **eTable 2.** Unit Costs for the Primary Analysis **eTable 3.** Unit Costs for Sensitivity Analyses **eTable 4.** Staff Mix of Surgeons in Theater **eTable 5.** Exploratory Analysis of Subgroup Differences by QALYs and Individual Components of Resource **eFigure 1.** Cost-Effectiveness Plane With Bootstrapped Incremental Costs and Quality-Adjusted Life-Years (QALYs) **eFigure 2.** Cost-Effectiveness Acceptability Curve for iRARC
eReferences

Supplement 2.
Nonauthor Collaborators

Supplement 3.
Data Sharing Statement
