# Supplementary material for: Cost-Effectiveness of Robot-Assisted Radical Cystectomy vs Open Radical Cystectomy for Patients With Bladder Cancer
Source: JAMA Netw Open. 2023 Jun 30;6(6):e2317255. doi: 10.1001/jamanetworkopen.2023.17255 (PMC10314306; doi:10.1001/jamanetworkopen.2023.17255)
Supplement: Supplement 1. — eMethods. Health Economics Analysis Plan eTable 1. Unit Cost of Robot per Patient eTable 2. Unit Costs for the Primary Analysis eTable 3. Unit Costs for Sensitivity Analyses eTable 4. Staff Mix of Surgeons in Theater eTable 5. Exploratory Analysis of Subgroup Differences by QALYs and Individual Components of Resource eFigure 1. Cost-Effectiveness Plane With Bootstrapped Incremental Costs and Quality-Adjusted Life-Years (QALYs) eFigure 2. Cost-Effectiveness Acceptability Curve for iRARC eReferences [file jamanetwopen-e2317255-s001.pdf]

Supplementary Online Content

Dixon S, Hill H, Flight L, et al; iROC Study Team. Cost-effectiveness of robot-assisted radical cystectomy vs open radical cystectomy for patients with bladder cancer. *JAMA Netw Open*. 2023;6(6):e2317255. doi:10.1001/jamanetworkopen.2023.17255

eMethods. Health Economics Analysis Plan

eTable 1. Unit Cost of Robot per Patient

eTable 2. Unit Costs for the Primary Analysis

eTable 3. Unit Costs for Sensitivity Analyses

eTable 4. Staff Mix of Surgeons in Theater

eTable 5. Exploratory Analysis of Subgroup Differences by QALYs and Individual Components of Resource

eFigure 1. Cost-Effectiveness Plane With Bootstrapped Incremental Costs and Quality-Adjusted Life-Years (QALYs)

eFigure 2. Cost-Effectiveness Acceptability Curve for iRARC

eReferences

This supplementary material has been provided by the authors to give readers additional information about their work.

© 2023 Dixon S et al. *JAMA Network Open*.

eMethods. Health Economics Analysis Plan

Contents

Section 1: Administrative Information ... 3
1.1 HEAP Administrative Information ... 3
Section 2: Trial Introduction & Background ... 5
2.1 Trial Background and Rationale ... 5
2.2 Aim of the Trial ... 5
2.3 Objectives of the trial ... 5
2.4 Trial Population ... 5
2.5 Intervention and Comparators ... 5
2.6 Trial Design ... 5
2.7 Trial Start and End Dates ... 5
Section 3: Economic Approach ... 6
3.1 Aims and Objectives of Economic Evaluation ... 6
3.3 Overview of Economic Analysis ... 6
3.4 Jurisdiction ... 6
3.5 Perspectives ... 6
3.6 Time Horizon ... 6
Section 4: Economic Data Collection and Management ... 6
4.1 Statistical Software use for Health Economic Analysis ... 6
4.2 Costing Approach ... 6
4.3 Measurement of Resource Use Data ... 6
4.4 Valuation of Resource Use Data ... 6
4.5 QALY Calculation Approach ... 7
4.6 Measurement of Outcomes ... 7
4.7 Valuations of Outcomes ... 7
Section 5: Economic Data Analysis ... 7
5.1 Analysis Population ... 7
5.2 Timing of Analyses ... 7
5.3 Discount Rates for Costs and Benefits ... 7
5.4 Cost-effectiveness Threshold(s) ... 7
5.7 Analysis of Costs ... 7
5.8 Analysis of Outcomes ... 7
5.9 Data Cleaning for Analysis ... 7
5.10 Missing Data ... 8
5.11 Analysis of Cost-effectiveness ... 8
5.12 Sampling Uncertainty ... 8
5.13 Subgroup Analyses/Analysis of Heterogeneity ... 8
5.14 Sensitivity Analyses ... 8
Section 7: Reporting/Publishing ... 9
7.1 Reporting Standards ... 9
7.2 Reporting Deviations from the HEAP ... 9
References ... 9

© 2023 Dixon S et al. *JAMA Network Open*.

Section 1: Administrative Information

1.1 HEAP Administrative Information

**Title**	Robot-assisted radical cystectomy with intracorporeal urinary diversion versus open radical cystectomy (iROC): health economic analysis
**Trial registration number; registry**	ISRCTN (13680280)<br>ClinicalTrials. gov (NCT03049410)
**Source of funding**	Champniss Foundation
**Purpose of HEAP**	The purpose of this HEAP is to describe the analysis and reporting procedure intended for the economic analysis to be undertaken. The analysis plan is designed to ensure that there is no conflict with the protocol and associated statistical analysis plan and it should be read in conjunction with them.
**Trial protocol version; date**	This document has been written based on information contained in the trial protocol version 4, dated 07/05/2019
**Trial Statistical Analysis Plan (SAP) version, date**	23 June 2020 DRAFT
**Trial HEAP version, date**	Version 1.0 23 July 2020
**HEAP revisions**	
**Roles and responsibilities**	This HEAP was prepared by Laura Flight (Health Economist) and approved by Prof Simon Dixon. The trial health economists are responsible for conducting and reporting the economic evaluation in accordance with the HEAP.

© 2023 Dixon S et al. *JAMA Network Open*.

Abbreviations and Definition

AE	Adverse Event
BC	Bladder Cancer
CEAC	Cost-effectiveness Acceptability Curve
CHEERS	Consolidated Health Economic Evaluation Reporting Standards
CI	Chief Investigator
CIS	Carcinoma in situ
CRF	Case Report Form
CUA	Cost-utility Analysis
DAOH	Days Alive and Out of Hospital
ECOG	Eastern Cooperative Oncology Group
eRARC	Extracorporeal Robot Assisted Radical Cystectomy
GBP	Great British Pounds
HRG	Healthcare Resource Groups
HRQoL	Health Related Quality of Life
ICER	Incremental Cost-Effectiveness Ratio
iRARC	Intracorporeal Robot assisted Radical Cystectomy
INMB	Incremental Net Monetary Benefit
ISRCTN	International Standard Randomised Controlled Trials Number
MDT	Multidisciplinary Team
MIBC	Muscle Invasive Bladder Cancer
NICE	National Institute for Health and Care Excellence
NHS	National Health Service
NMIBC	Non-muscle Invasive Bladder Cancer
ORC	Open Radical Cystectomy
PET	Positron Emission Tomography
PI	Principal Investigator
PSSRU	Personal Social Services Research Unit
QALY	Quality Adjusted Life Years
RC	Radical Cystectomy
RARC	Robot Assisted Radical Cystectomy
ROC	Receiver Operating Characteristic
SAP	Statistical Analysis Plan
SCC	Squamous Cell Carcinoma
SITU	Surgical & Interventional Trials Group
TCC	Transitional Cell Carcinoma of Bladder
TMG	Trial Management Group
TNM	The TNM Classification of Malignant Tumours
TSC	Trial Steering Committee
TURBT	Trans Urethra Resection of Bladder Tumour
UCC	Urothelial Cell Carcinoma
UCL	University College London
UK	United Kingdom

© 2023 Dixon S et al. *JAMA Network Open*.

Section 2: Trial Introduction & Background

2.1 Trial Background and Rationale

Bladder cancer (BC) is a common malignancy and one of the most expensive to manage [**Error! Reference source not found.**, **Error! Reference source not found.**]. Radical cystectomy (RC) represents the gold standard treatment and plays a key role in managing high-risk non-muscle invasive cancer and in salvage after radiotherapy [**Error! Reference source not found.**]. RC is a major operation associated with frequent comorbidity [**Error! Reference source not found.**, **Error! Reference source not found.**]. Reductions in morbidity and mortality could be achieved through robot-assisted surgery, however, there is little or conflicting evidence of benefit from robot-assisted RC (RARC) over the open approach (ORC), [**Error! Reference source not found.-Error! Reference source not found.**] and there are uncertainties about oncological outcomes with robotic surgery [**Error! Reference source not found.**].

The principle advantages of robotic radical cystectomy to the participant and the health system are thought to be:

1. A reduced length of hospital stay due to less invasive surgery, reduced need for blood and reduced complications.
2. Reduced re-admissions up to 90 days post-surgery due to reduced complications and associated adverse events.
3. Improved health-related quality of life owing to the above effects, plus faster return to normal functioning.

However, these advantages need to be carefully assessed against the higher costs of robotic surgery that include the capital and consumable costs.

2.2 Aim of the Trial

The iROC trial prospectively randomised eligible participants to either robot-assisted radical cystectomy (iRARC) or open radical cystectomy (ORC) and measured their recovery in order to compare oncological outcomes, safety, and cost-effectiveness and determine the role of both approaches in participant care.

2.3 Objectives of the trial

The primary clinical objective was to compare the number of days alive and out of hospital (DAOH) within 90 days from surgery. Secondary objectives assessed recovery, complications, oncological outcomes and surgeon fatigue measured up to 12 months. Quality of life outcomes are assessed up to 78 weeks from surgery. Data up to 90 days are used in this analysis.

2.4 Trial Population

The trial aims to recruit 340 participants. An internal feasibility study was conducted after the first 30 patients. [**Error! Reference source not found.**]

Inclusion Criteria:

1. Participants must be over 18 years of age.
2. Histopathological confirmation of BC (Urothelial Cell Carcinoma (UCC), Squamous Cell Carcinoma (SCC), adenocarcinoma or rare variant).
3. Carcinoma in situ (CIS) or stage pTa or pT1 or ≥pT2 or mobile bladder mass on bimanual examination under anaesthesia.
4. Node status ≤N1 on imaging criteria or positron emission tomography (PET)–ve outside pelvis.
5. Eastern Cooperative Oncology Group (ECOG) grade 1, 2 or 3.
6. Able to give informed written consent to participate.

Exclusion Criteria:

1. Unwilling to undergo cystectomy.
2. Previous abdominal surgery rendering them unsuitable for either iRARC or ORC.
3. Participants with upper urinary tract disease.
4. Concomitant disease that would render the participant unsuitable for the trial.
5. Pregnant or lactating females.
6. Previous radiotherapy for BC.

2.5 Intervention and Comparators

Participants were randomised using a 1:1 allocation ratio to

1. *Intervention:* intracorporeal robot-assisted radical cystectomy (iRARC)
2. *Comparator*: open radical cystectomy (ORC)

2.6 Trial Design

iROC is a prospective multicentre randomised controlled trial in participants undergoing RC for BC. Eligible participants were consented and randomised to iRARC or ORC after multidisciplinary team (MDT) review in National Health Service (NHS) cancer centres. Due to the nature of the interventions, it was not possible to blind the participants or staff to treatment allocations.

2.7 Trial Start and End Dates

The trial opened to recruitment in March 2017 using protocol version 1.0 (dated November 2016) and was due to close in August 2020 or after accrual and the last participant 90 day assessments are completed. Follow-up of participants will continue until December 2021 to collect data on secondary outcomes.

© 2023 Dixon S et al. *JAMA Network Open*.

Recruitment closed in March 2020 due to COVID-19 at 339 participants.

Section 3: Economic Approach

3.1 Aims and Objectives of Economic Evaluation

The primary objective of the health economic evaluation is to estimate the short-term cost-effectiveness of iRARC compared to ORC for the radial cystectomy of participants with bladder cancer.

3.3 Overview of Economic Analysis

The study will follow good practice guidelines for economic evaluations undertaken alongside controlled trials [**Error! Reference source not found.**]. In summary, resource use and health outcomes will be measured for each p articipant in the trial. Costs and quality adjusted life years (QALYs) will then be calculated for each individual participant. Differences between the two arms will be calculated and cost-effectiveness compared against funding thresholds used by the National Institute for Health and Care Excellence (NICE).

3.4 Jurisdiction

The trial is being conducted in the United Kingdom (UK) in the NHS setting.

3.5 Perspectives

The primary health economic analysis will be from the NHS perspective.

3.6 Time Horizon

The economic analysis will be a within trial analysis using data from the 90 day follow-up of trial participants.

Section 4: Economic Data Collection and Management

4.1 Statistical Software use for Health Economic Analysis

The latest versions of R [**Error! Reference source not found.**] and Stata [**Error! Reference source not found.**] wi ll be used for analysis.

4.2 Costing Approach

The costing approach will include identification of resource use, measurement and valuation [14]. Costs will include (but are not limited to):

- Procedure, including;
    - Theatre equipment cleaning & sterilization
    - Robotic disposables
    - Theatre consumables,
    - Theatre time,
    - Theatre staff costs,
    - Overheads,
    - Service charge,
    - Training of surgeons.
- Post-operative hospital stay
- Blood transfusions during post-operative stay
- GP visits
- Emergency department visits
- Associated adverse events
- Length of stay in critical care
- Readmissions
- Conversion costs from iRARC to open ORC
- Conversion from intra-corporeal to extra-corporeal reconstruction

4.3 Measurement of Resource Use Data

For this analysis, resource use data will be collected for the 90 days following surgery using case report forms (CRFs), completed by healthcare professionals. To capture all relevant aspects of resource use in the trial the following case report forms are used:

- Cystectomy Form (day 0)
- Inpatient Stay Form (discharge day 7-14)
- Blood Transfusion Form
- Adverse Events/ Surgical Complications Form
- Outpatient Follow-Up Form (3-months)

The CRFs asked participants to indicate the number of events, such as number of visits to the GP however, the duration of each visit was not recorded and therefore average estimates will be used based on the NHS national reference costs or the costs of health and social care, where relevant [15,16]

© 2023 Dixon S et al. *JAMA Network Open*.

4.4 Valuation of Resource Use Data
The unit costs of each procedure will be estimated using a consistent approach to allow for a fair comparison. The costs of the initial procedure will be based on a cost for the robot (if appropriate), cost per theatre minute and cost per length of stay derived from financial data from the largest recruiting centres. In addition to capital, equipment, staff costs and overheads, and the costs required to train surgeons to undertake robotic assisted surgery will be estimated using expert opinion.
Costs of subsequent procedures within the same hospital stay will be calculated as the Reference Cost for that procedure minus the ward costs for that procedure; ‘ward costs’ calculated as the excess bed day cost for the HRG multiplied by the average length of stay for that HRG.
Costs of readmissions will be based on Reference Costs for their associated HRGs. Costs of blood products will be come from NHS Blood and Transplant.
The remaining items of resource use will be valued using appropriate unit costs from standard health economic sources such as

- British National Formulary [17].
- NHS Reference Costs [16].
- PSSRU Unit Costs of Health and Social Care [**Error! Reference source not found.**].

Costs are calculated using Great British pounds (GBP) for the most recent year for which the major source of cost difference between the arms is expected to be (here the cost of the intervention), where costs are not available for this year they will be inflated using the NHS Cost Inflation Index for pay and prices [**Error! Reference source n ot found.**].
4.5 QALY Calculation Approach
The primary health economic outcome measure will be the QALY. This combines both the quality and quantity of life and is measured using utility [**Error! Reference source not found.**]. Utility quantifies health related quality o f life (HRQoL) and is a value anchored between zero and one, where one represents full health and zero represents death. Utilities below zero represent states worse than death. Utilities will be derived from the EQ-5D-5L questionnaire, a self-completion tool for participants, that is applicable to a wide range of health conditions and treatments [**Error! Reference source not found.**]. Measured domains include mobility, self-care, usual activities, pain and anxiety or depression.
4.6 Measurement of Outcomes
The EQ-5D-5L will be completed by participants at baseline, 5 weeks, 12 weeks, 26 weeks, 52 and 78 weeks. Data at 90 days (12 weeks) will be used in this analysis.
4.7 Valuations of Outcomes
The EQ-5D-5L health states will be converted on to the three level scale (EQ-5D-3L), and utility scores will be derived using the UK tariff, as this is preferred by NICE, using the crosswalk [20, 21].
During the first month of the trial each participant will undergo surgery. It is anticipated during this time the utility between each intervention arm may differ given the potential impact on recovery rates from the new procedure. Using linear interpolation between baseline and one-month follow-up, to calculate the QALY is unlikely to be sensitive to differences in HRQoL of participants during this time. Therefore data collected between baseline and one-month follow-up on activity levels will be used to calculate a more accurate QALY.
Quantified activity level is collected at baseline, Day 0, day five, discharge, one and three months. Fitness tracking devices will record steps taken for seven consecutive days at predetermined time points. This will be used impute EQ-5D data at day five post-operative using covariates such as age, gender and one month EQ-5D. The imputed day 5 post-operative EQ-5D will then be used alongside baseline, one month and three month EQ-5D scores to calculate the QALY (using linear interpolation). Alternative approaches for calculating the QALY are explored in sensitivity analyses.

Section 5: Economic Data Analysis

5.1 Analysis Population
The full analysis set for the health economic analysis will include all participants randomised, known as the intention-to-treat population.
5.2 Timing of Analyses
The primary health economic analysis will be conducted once the final patient has completed the 3-month follow-up of the study.
5.3 Discount Rates for Costs and Benefits
Capital costs of equipment will be annuitized over its lifespan using a discount rate of 3.5%. Resource costs and benefits will not be discounted as the trial data only cover a 3-month period.
5.4 Cost-effectiveness Threshold(s)
A range of willingness to pay thresholds will be considered including the NICE threshold of £20,000 per QALY.

© 2023 Dixon S et al. *JAMA Network Open*.

5.7 Analysis of Costs

Histograms of total costs for complete case data will be used to visualise the distribution of cost data. Mean total costs in each arm will be summarised and then broken down into the different components of resource use to identify the drivers in the total costs.

5.8 Analysis of Outcomes

Mean utility at each time point will be plotted visually using a line plot to illustrate the pattern of QALY over the trial period for each intervention. Mean QALY will be calculated using linear interpolation between time points.

5.9 Data Cleaning for Analysis

Data will be checked for face validity and any unusual results queried with the study team. Any changes to the data will be documented and implemented using R code. No changes will be made to the original data.

5.10 Missing Data

Missing data can give misleading estimates of a within-trial cost-effectiveness analysis. A complete-case analysis uses only participants with no missing data in the key cost and benefit outcomes. This is undesirable as it reduces the sample size and affects the power of the study [22]. To handle missing data from the trial, the following assumptions will be made:

- If someone dies during the study their subsequent quality of life measures will be set to zero and all future costs will be set to zero.
- If someone withdraws from the study any data collected at their withdrawal meeting will be assigned to the next nearest time point as appropriate.

Using the recommendations for Faria *et* al (2014) [**Error! Reference source not found.**] patterns of missing data w ill be assessed in the data including

1. Proportion of missing data by treatment arm, at each follow-up period, to assess whether or not missing data differed by arm.
2. Missing data patterns to determine whether or not data were missing for all items or individual items of utility scores and resource use items over the trial follow-up.

If deemed to be appropriate, the multiple imputation chained equation method with predictive mean matching will be used to impute missing values of costs, QALYs and baseline covariates. The number of imputations will be based on the highest percentage of missing data for the variables of interest (baseline utility, QALYs and total cost). The imputation will be performed per randomisation arm, for all imputed variables, except baseline covariates with missing data, for which imputation will be performed across all observations.

5.11 Analysis of Cost-effectiveness

Cost-utility analysis (CUA) will be performed to compare the cost-effectiveness of iRARC to ORC. A regression model will be used to estimate the difference in mean total costs and QALYs between treatment arms, taking into account the correlation between total costs and QALYs [23]. Model parameter estimates will be used to address uncertainty [**Error! Reference source not found.**].

The regression equation for total costs will include the randomisation arm. The regression equation for QALY will include the randomisation arm and baseline utility to control for imbalances in baseline utility between treatment arms [23, 25].

The incremental cost-effectiveness ratio (ICER) will be estimated from model output and compared with the NICE cost-effectiveness threshold of £20,000 per QALY gained.

5.12 Sampling Uncertainty

To illustrate uncertainty cost-effectiveness confidence ellipses and net monetary benefit (NMB) lines with confidence intervals (CI) will be produced. The willingness to pay threshold will be varied to assess the uncertainty associated with the estimates. Additionally, a cost-effectiveness acceptability curve (CEAC) will be constructed illustrating the probability of each treatment being most cost-effective for a range of threshold values.

5.13 Subgroup Analyses/Analysis of Heterogeneity

The analysis will be repeated focussing on the following subgroups

- Chemotherapy versus no chemotherapy
- Stage ≤T2 vs ≥T3
- Age greater than 70 years
- Performance status 0 vs ≥1
- Gender
- Type of diversion for example Stoma/neo-bladder

5.14 Sensitivity Analyses

A number of sensitivity analyses will be performed to assess the robustness of the within-trial health economic estimates. In each case a similar regression model will be used to estimate differences in total costs, differences in QALYs and the ICER. The analyses include:

1. Complete case analysis including patients with complete data.
2. Using a willingness to pay threshold of £30,000 per QALY.

© 2023 Dixon S et al. *JAMA Network Open*.

3. QALYs will be calculated using alternative specifications such as using the alternative crosswalk tariffs for the EQ-5D-5L
4. Lifetime time horizon will be explored using alternative specifications of the risk of death following surgery, for example considering whether the difference in risk between the two arms is maintained over time and using different sources of data relating to life expectancy.
5. Scenario analysis to reflect any changes that might occur as staff become more experienced for example, less time and less staff members might needed.
6. The costs incurred by a patient per day are not linear over their total hospital stay. Large costs are likely to be incurred at the start of the stay when they receive the surgery and fewer costs incurred in the days immediately before discharge. As iRARC is thought to reduce the number of days spent in hospital, it is important to think about the cost of the days that are saved. The costing procedure described previously values hospital days using a ward day cost based on largest recruiting sites. The sensitivity analysis will explore alternative estimates of the cost of the days saved by the new intervention.
7. Uncertainties in the lifespan and throughput of the new intervention will be explored.

Section 7: Reporting/Publishing

7.1 Reporting Standards

The results of the within trial analysis will be reported in line with the CHEERS checklist for reporting economic evaluations. [**Error! Reference source not found.**]
This HEAP was written using the template provided by Dr Joanna Thorn (February 2019) [27].

7.2 Reporting Deviations from the HEAP

Any deviation from HEAP will be described and justified in the final published report.

References

1. Antoni S, Ferlay J, Soerjomataram I, et al. Bladder cancer incidence and mortality: a global overview and recent trends. Eur Urol 2017;71:96–108.
2. Svatek RS, Hollenbeck BK, Holmäng S, et al. The economics of bladder cancer: costs and considerations of caring for this disease. Eur Urol 2014;66:253–62.
3. Babjuk M, Böhle A, Burger M, et al. EAU Guidelines on Non-Muscleinvasive Urothelial Carcinoma of the Bladder: Update 2016. Eur Urol 2017;71:447–61.
4. Shabsigh A, Korets R, Vora KC, et al. Defining early morbidity of radical cystectomy for participants with bladder cancer using a standardized reporting methodology. Eur Urol 2009;55:164–74.
5. Tan WS, Lamb BW, Tan MY, et al. In-depth Critical Analysis of Complications Following Robot-assisted Radical Cystectomy with Intracorporeal Urinary Diversion. Eur Urol Focus 2017;3:30062–1.
6. Tan WS, Sridhar A, Ellis G, et al. Analysis of open and intracorporeal robotic assisted radical cystectomy shows no significant difference in recurrence patterns and oncological outcomes. Urol Oncol 2016;34:257.e1–257.e9.
7. Yuh B, Wilson T, Bochner B, et al. Systematic review and cumulative analysis of oncologic and functional outcomes after robot-assisted radical cystectomy. Eur Urol 2015;67:402–22.
8. Wilson TG, Guru K, Rosen RC, et al. Best practices in robot-assisted radical cystectomy and urinary reconstruction: recommendations of the Pasadena Consensus Panel. Eur Urol 2015;67:363–75.
9. Hussein AA, Saar M, May PR, et al. Early Oncologic Failure after Robot-Assisted Radical Cystectomy: Results from the International Robotic Cystectomy Consortium. J Urol 2017;197:1427–36.
10. Catto JW, Khetrapal P, Ambler G, et al. Multidomain quantitative recovery following radical cystectomy for patients within the robot-assisted radical cystectomy with intracorporeal urinary diversion versus open radical cystectomy randomised controlled trial: the first 30 patients. European urology. 2018;74(4):531-4.
11. Ramsey S, Willke R, Glick H, et al. Cost-Effectiveness Analysis Alongside Clinical Trials II: An ISPOR Good Research Practices Task Force Report. Value in Health 2015;18(2):161-172.
12. R Core Team (2018). R: A language and environment for statistical computing. R Foundation for Statistical Computing, Vienna, Austria. URL https://www.R-project.org/.
13. StataCorp. 2017. Stata Statistical Software: Release 15. College Station, TX: StataCorp LLC
14. Drummond M, Brandt A, Luce B, et al. Standardizing methodologies for economic evaluation in health care. Int J Technol Assess Health Care. 1993; 9(1) :26–36
15. Curtis, Lesley A. and Burns, Amanda (2018) Unit Costs of Health and Social Care 2018. Project report. University of Kent https://doi.org/10.22024/UniKent/01.02.70995

© 2023 Dixon S et al. *JAMA Network Open*.

16. Department of Health. Reference Costs 2017/18. 2018. https://improvement.nhs.uk/resources/reference-costs/
17. Joint Formulary Committee. British National Formulary (online) London: BMJ Group and Pharmaceutical Press Available from http://www.medicinescomplete.com. Last accessed 13th May 2019
18. Drummond MF, Sculpher MJ, Claxton K, et al. Methods for the economic evaluation of health care programmes. Oxford university press; 2015 Sep 25.
19. Herdman M, Gudex C, Lloyd A, et al Development and preliminary testing of the new five-level version of EQ-5D (EQ-5D-5L). Quality of life research. 2011 Dec 1;20(10):1727-36.
20. van Hout B, Janssen MF, Feng YS, et al. Interim scoring for the EQ-5D-5L: mapping the EQ-5D-5L to EQ-5D-3L value sets. Value in health. 2012 Jul 1;15(5):708-15.
21. NICE. Position statement on use of the EQ-5D-5L valuation set. 2017; https://www.nice.org.uk/Media/Default/About/what-we-do/NICE-guidance/NICE-technology-appraisal- guidance/eq5d5l_nice_position_statement.pdf. Accessed: 16.2.2019
22. Faria R, Gomes M, Epstein D, et al. A guide to handling missing data in cost-effectiveness analysis conducted within randomised controlled trials. Pharmacoeconomics. 2014; 32: 1157–70.
23. Willan AR, Briggs AH, Hoch JS. Regression methods for covariate adjustment and subgroup analysis for non-censored cost-effectiveness data. Health Econ. 2004;13:461–75.
24. Briggs AH. Handling uncertainty in economic evaluation and presenting the results. In: Drummond M, McGuire A, editors. Economic evaluation in health care. Merging theory with practice. Oxford, New York: Oxford University Press; 2001.
25. Manca A, Hawkins N, Sculpher MJ. Estimating mean QALYs in trial-based cost-effectiveness analysis: the importance of controlling for baseline utility. Health Econ. 2005;14:487–96.
26. Husereau D, Drummond M, Petrou S, et al Consolidated health economic evaluation reporting standards (CHEERS) statement. Cost Effectiveness and Resource Allocation. 2013 Dec;11(1):6.
27. Thorn JC, Ridyard C, Hughes D, et al. (2017) 'Health economics analysis plans: the current state of play' *Trials*, 18(Suppl 1):P144

eTable 1. Unit Cost of Robot per Patient

Cost component	Value (£)	Source	Derivation
Da Vinci X	Confidential	Contract price provided by study center.	Inflated to 2020/21 prices using the NHSCII. [1] Annuitized over 10 years at 3.5% in advance, with zero residual value and a throughput for all types of surgery of 207 patients per annum. Throughput based on Lam et al. [2] but author contacted for exact number that could only be estimated from a graph.
Annual maintenance	Confidential	Contract price provided by study center, inflated to 2020/21 prices using the NHSCII	Allocated using throughput for all types of surgery of 207 patients per annum. Throughput derived as above.
Simulator price	Not reported to prevent derivation of confidential prices	Provided following request to Intuitive Surgical	Annuitized at 3.5% in advance, and a throughput for all types of surgery on 207 patients per annum. Throughput derived as above.
Instruments	Not reported to prevent derivation of confidential prices	Provided following request to Intuitive Surgical.	Relates to costs via the Intuitive Surgical Extended Use Programme. The items of equipment included represents the most common set of instruments used in the UK. These were; large needle driver (x2), Prograsp forceps, Maryland bipolar forceps, MCS and small grasping retractor.
Training	22 [$32]	Based on Intuitive surgical requirements and study center estimate of mentoring time.	Based on online training (2.5hrs), simulation training (35hrs), wet lab training (15hrs), mentoring (13.1hrs) and a consultant surgeon cost per hour of £114 [$165] (Jones 2021). Annuitized over 10 years at 3.5% in advance, with zero residual value and robot-associated workload of 40 cases per surgeon per annum. Travel and accommodation for training was provided by Intuitive Surgical, therefore, £0 [$0] from the NHS perspective.
**Total**	**2638 [$3807]**		

© 2023 Dixon S et al. *JAMA Network Open*.

eTable 2. Unit Costs for the Primary Analysis

Resource	Unit cost (£, 2019/20)	Source	Derivation
Robot per patient	2638 [$3807]	See Supplement 2	See Supplement 2
Open radical equipment per patient	1514 [$2185]	Bansal (2018)[3]	No financial year is given and so the published figure is used unadjusted.
Ward day	518 [$747]	Study center	Derived from a breakdown of National Reference Costs for LB39C and LB39D at one study center, adjusted to reflect national average costs using NHS England (2019/20) [4]
Cost per minute of theater (consultant)	35 [$50]	Study center	Derived from a breakdown of theater costs at one study center, adjusted to reflect national average costs
Cost per minute of theater (consultant with trainee)	38 [$55]	Study center	
Cost per minute of theater (two consultants)	43 [$62]	Study center	
Intensive therapy unit day (respiratory failure)	1266 [$1827]	NHS England (2019/20) [4]	Cost relates to unspecified surgical specialty 1 organ supported
Intensive therapy unit day (other)	1863 [$2688]	NHS England (2019/20) [4]	Cost relates to unspecified surgical specialty 2-6 organs supported, calculated as an activity weighted mean cost
HDU day	579 [$835]	NHS England (2019/20) [4]	Estimated as non-standard location on a ward area, 0 organs supported
Readmission day	582 [$840]	NHS England (2017/18) [5]	Calculated using all HRGs, non-elective long stay admission, using inlier total costs and bed days from 2017/18 National Reference Costs, inflated using NHS Cost and Inflation Index. [1]
Unit of blood	133 [$192]	NHS Blood and Transplant Price List 2019/20 (available only on request to NHS Blood and Transplant)	Price for a unit of Standard Red Cells
Family physician visit	39 [$56]	Curtis 2020 [6]	Cost per consultation including direct care staff costs and qualifications.
Emergency department attendance	165 [$238]	NHS England (2019/20) [4]	Category 1 Investigation with Category 1-2 Treatment, Type 1 Unit.

© 2023 Dixon S et al. *JAMA Network Open*.

eTable 3. Unit Costs for Sensitivity Analyses

Resource and description	Primary analysis (£, 2019/20)	Lower value (£, 2019/20)	Upper value (£, 2019/20)	
Lower theater cost per minute for iRARC to reflect the potential for shorter surgery or changes in staff mix for iRARC	35 [$50] (C only)<br>38 [$55] (C+trainee)<br>43 [$62] (2C)	31 [$45]<br>35 [$50]<br>39 [$56]	33 [$48]<br>36 [$53]<br>41 [$59]	5% and 10% reductions in theater costs used in the primary analysis for iRARC
Lower ORC equipment costs to reflect other published estimates	1514 [$2185]	757 [$1902]	1136 [$1639]	Other published estimates are $1254, £638 and $167 (disposables).[7-9] To reflect these, the estimate used in the primary analysis reduced to 75% and 50% of its value.
Ward day; lower value to reflect marginal cost of saved days (as opposed to the average cost used in the primary analysis)	518 [$747]	335 [$483]	Not applicable	Lower value calculated by removing the overhead component of the ward costs provided by the study center as these are unlikely to vary with reduced length of stay.
Robotic surgery; shorter longevity of use with lower throughput (upper value), and longer longevity of use with higher throughput (lower value)	2638 [$3807]	2310 [$3333]	3341 [$4821]	Upper value calculated using a reduced machine life expectancy of 7 years together with a 25% reduction in throughput. Lower value calculated using a machine life expectancy of 13 years together with a 25% increase in throughput.
Cost per minute of theater and ward cost; upper and lower values to capture variation between hospitals	35 [$50] (C only)<br>38 [$55] (C+trainee)<br>43 [$62] (2C)<br>518 [$747] (ward day)	20 [$29]<br>22 [$31]<br>25 [$36]<br>295 [$426]	43 [$61]<br>47 [$67]<br>53 [$76]<br>632 [$912]	Upper value adjusted to upper quartile NHS costs. Lower value adjusted to lower quartile NHS costs.

Abbreviations: iRARC, intracorporeal robot-assisted radical cystectomy; ORC, open radical cystectomy; NHS, National Health Service; C, consultant surgeon; 2C, two consultant surgeons.

© 2023 Dixon S et al. *JAMA Network Open*.

eTable 4. Staff Mix of Surgeons in Theater

	Open radical cystectomy	Intracorporeal robot-assisted radical cystectomy	Row totals
	Observed (Expected) [chi-squared statistic]		
Consultant only	16 (26.78) [4.34]	39 (28.22) [4.11]	55
Consultant plus registrar	104 (95.91) [0.68]	93 (101.09) [0.65]	197
Consultant plus consultant	25 (22.39) [0.30]	21 (23.61) [0.29]	46
Unknown	3 (2.92) [0.00]	3 (3.08) [0.00]	6
Column totals	148	156	304

X^2 (3, N = 304) = 10.3769, p = .02.

© 2023 Dixon S et al. *JAMA Network Open*.

eTable 5. Exploratory Analysis of Subgroup Differences by QALYs and Individual Components of Resource

Resource	ORC	iRARC	Increment	p-value of increment*	p-value of equality of increments across sub-groups**
**Age**					
**Age greater than 70 years**					
Theater minutes, mean (SD)	258.6	290.8	- 32.2	.01	.94
Ward days, mean (SD)	11.87	8.56	3.31	.02	.03
No. admitted to ITU (%)	8	3	5	.06	.47
Readmission (%)	20	7	13	.46	.03
QALYs	0.18658	0.19985	0.01327	.01	.63
**Age less than 70 years**					
Theater minutes, mean (SD)	275.1	308.6	- 33.50	.01	.94
Ward days, mean (SD)	8.66	9.18	- 0.52	.62	.03
No. admitted to ITU (%)	8	4	4	.31	.47
Readmission (%)	27	20	7	< .01	.03
QALYs	0.17759	0.18801	0.01042	.04	.63
**Tumor stage**					
**Stage ≥T3**					
Theater minutes, mean (SD)	272.3	301.6	- 29.3	.04	.80
Ward days, mean (SD)	14.4	8.7	5.6	.02	.03
No. admitted to ITU (%)	4	3	1	.51	.58
Readmission (%)	9	7	2	.33	.48
QALYs	0.18396	0.18912	0.00516	.50	.40
**Stage ≤T2**					
Theater minutes, mean (SD)	264.9	298.7	- 33.85	< .01	.80
Ward days, mean (SD)	8.79	9.01	- 0.22	.80	.03
No. admitted to ITU (%)	11	4	7	.07	.58
Readmission (%)	35	16	19	< .01	.48
QALYs	0.18057	0.19352	0.01295	.01	.40
**Performance status**					
**Performance status ≥1**					
Theater minutes, mean (SD)	251.18	267.39	- 16.20	.48	.52
Ward days, mean (SD)	11.88	9.44	2.44	.39	.70
No. admitted to ITU (%)	5	1	4	.13	.38
Readmission (%)	8	5	3	.38	.90
QALYs	0.17397	0.19289	0.01892	.03	.23
**Performance status is 0**					
Theater minutes, mean (SD)	271.78	304.25	- 32.47	< .01	.52
Ward days, mean (SD)	9.70	8.43	1.27	.17	.70
No. admitted to ITU (%)	11	6	5	.22	.38
Readmission (%)	35	21	14	.04	.90
QALYs	0.18438	0.19509	0.01071	.01	.23

* This relates to the difference between treatment arms within the subgroup.

© 2023 Dixon S et al. *JAMA Network Open*.

** This tests whether the increments between the sub-groups are statistically different e.g. tests if the increments (ORC versus iRARC) are the same between one group (the ages above 70) and the other (below 70 years age). The p-values are replicated for each subgroup for ease of reference.

The tests of equality of increments are undertaken using a with split-sample regression. That is, a regression is run in one subgroup (e.g. patients with a performance status of zero) and a statistical test (Chi squared) is used to determine if the treatment arm variable (for each cost component and QALY) is the same as when the regression is run on the other subgroup (e.g. performance status is one or above). The split sample regressions applied ordinary least squares when the dependent variable is continuous and logit regression when the dependent variable is binary.

The figures in the columns to the left of that test also come from the split sample regression, except for QALYs. The preceding figures for QALYs are taken from Table 3 in the manuscript for consistency, which were generated from a seemingly unrelated regression of costs and QALYs, with sub-groups incorporated using interaction terms. This approach is not possible with the analysis of multiple components of resource.

© 2023 Dixon S et al. *JAMA Network Open*.

eFigure 1. Cost-Effectiveness Plane With Bootstrapped Incremental Costs and Quality-Adjusted Life-Years (QALYs)

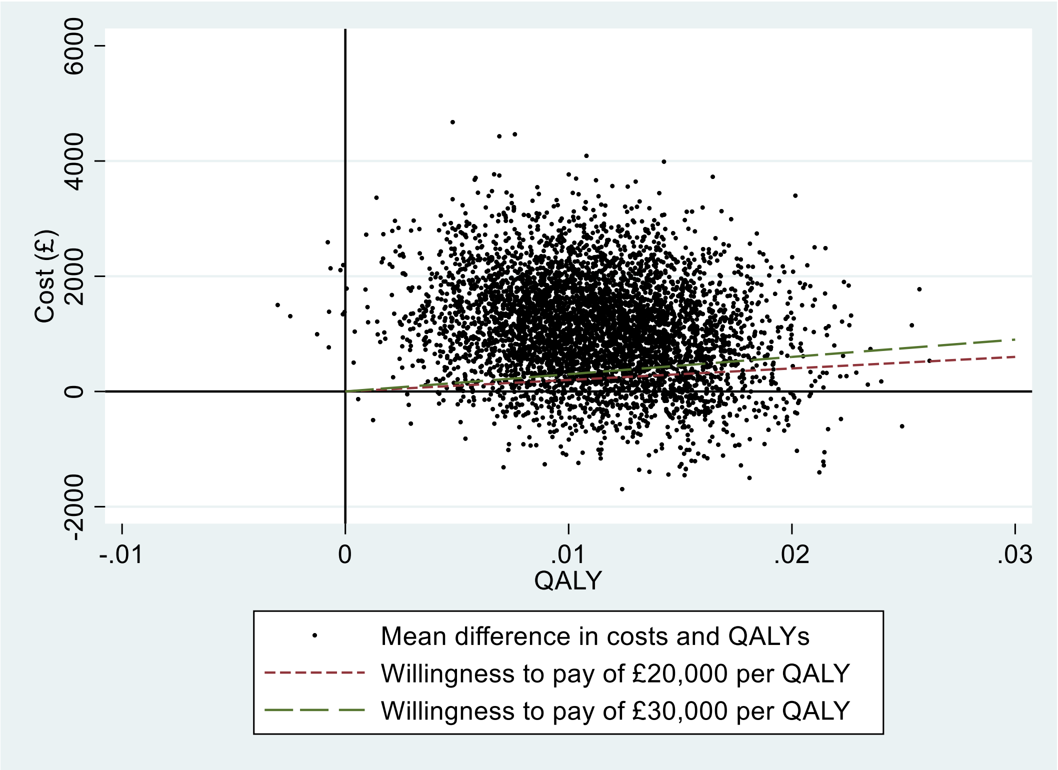

© 2023 Dixon S et al. *JAMA Network Open*.

eFigure 2. Cost-Effectiveness Acceptability Curve for iRARC

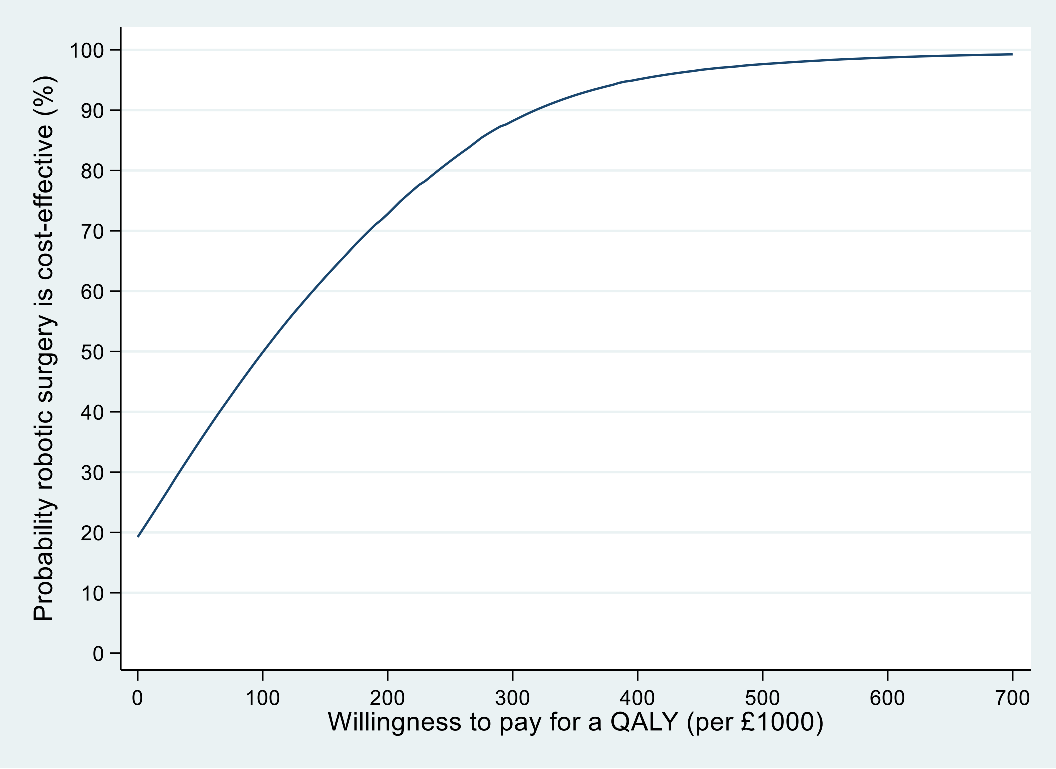

QALY = quality adjusted life year

© 2023 Dixon S et al. *JAMA Network Open*.

eReferences

1. Jones KC, Burns A. *Unit Costs of Health and Social Care 2021*. Unit Costs of Health and Social Care. Personal Social Services Research Unit, University of Kent; 2021.

2. Lam K, Clarke J, Purkayastha S, Kinross JM. Uptake and accessibility of surgical robotics in England. *Int J Med Robot*. Feb 2021;17(1):1-7. doi:10.1002/rcs.2174

3. Bansal SS, Dogra T, Smith PW, et al. Cost analysis of open radical cystectomy versus robot-assisted radical cystectomy. *BJU Int*. Mar 2018;121(3):437-444. doi:10.1111/bju.14044

4. NHS England. NHS Reference Costs 2019/20. Accessed 4th October, 2022. https://www.england.nhs.uk/publication/2019-20-national-cost-collection-data-publication/

5. NHS England. NHS Reference Costs 2017/18. Accessed 4th October, 2022. https://webarchive.nationalarchives.gov.uk/ukgwa/20200501111106/https://improvement.nhs.uk/resources/reference-costs/

6. Curtis LA, Burns A. *Unit Costs of Health and Social Care 2020*. Unit Costs of Health and Social Care. Personal Social Services Research Unit, University of Kent; 2020.

7. Hermans TJ, Fossion LM. What about conventional laparoscopic radical cystectomy? Cost-analysis of open versus laparoscopic radical cystectomy. *J Endourol*. Apr 2014;28(4):410-5. doi:10.1089/end.2013.0550

8. Lee R, Ng CK, Shariat SF, et al. The economics of robotic cystectomy: cost comparison of open versus robotic cystectomy. *BJU Int*. Dec 2011;108(11):1886-92. doi:10.1111/j.1464-410X.2011.10114.x

9. Labban M, Dasgupta P, Song C, et al. Cost-effectiveness of Robotic-Assisted Radical Prostatectomy for Localized Prostate Cancer in the UK. *JAMA Netw Open*. Apr 1 2022;5(4):e225740. doi:10.1001/jamanetworkopen.2022.5740

© 2023 Dixon S et al. *JAMA Network Open*.
