# Supplementary material for: Cost-Effectiveness of Robot-Assisted Radical Cystectomy vs Open Radical Cystectomy for Patients With Bladder Cancer
Source: JAMA Netw Open. 2023 Jun 30;6(6):e2317255. doi: 10.1001/jamanetworkopen.2023.17255 (PMC10314306; doi:10.1001/jamanetworkopen.2023.17255)
Supplement: Supplement 2. — Nonauthor Collaborators [file jamanetwopen-e2317255-s002.pdf]

Supplemental Online Content: Nonauthor Collaborators
*First name, last name, and suffix (if applicable) are required and will appear in PubMed.

*Group Name(s): iROC Study Team							
*First Name and Middle Initial(s)	*Last Name	*Suffix (eg, Jr, III)	Academic Degrees	Institution	Location (city, state/province, country)	Role or Contribution, eg, chair, principal investigator	Group (if more than 1 Group listed in the byline) and/or Subgroup (eg, Steering Committee)
E Ruth	Groves			Sheffield Teaching Hospital NHS Fou	Sheffield, UK	Medical staff involved in patient care	
Louise	Goodwin			Sheffield Teaching Hospital NHS Fou	Sheffield, UK	Research Nurse	
Jayne	Willson			Sheffield Teaching Hospital NHS Fou	Sheffield, UK	Research Nurse	
Phillip	Ravencroft			Sheffield Teaching Hospital NHS Fou	Sheffield, UK	Research Nurse	
Stephen	Kennish			Sheffield Teaching Hospital NHS Fou	Sheffield, UK	Medical staff involved in patient care	
Derek J	Rosario			Sheffield Teaching Hospital NHS Fou	Sheffield, UK	Medical staff involved in patient care	
Carol	Torrington			Sheffield Teaching Hospital NHS Fou	Sheffield, UK	Admin and secretarial support	
Rajesh	Nair			Guys and St Thomas' NHS Foundatio	London, UK	Medical staff involved in patient care	
Ramesh	Thurairaja			Guys and St Thomas' NHS Foundatio	London, UK	Medical staff involved in patient care	
Sue	Amery			Guys and St Thomas' NHS Foundatio	London, UK	Bladder cancer clinical nurse specialist	
Kathryn	Chatterton			Guys and St Thomas' NHS Foundatio	London, UK	Bladder cancer clinical nurse specialist	
Samantha	Broadhead			Guys and St Thomas' NHS Foundatio	London, UK	Research Nurse	
David	Hendry			NHS Greater Glasgow and Clyde	Glasgow, UK	Medical staff involved in patient care	
Abdullah	Zreik			NHS Greater Glasgow and Clyde	Glasgow, UK	Medical staff involved in patient care	
Sunjay	Jain			Leeds Teaching Hospitals NHS Trust	Leeds, UK	Medical staff involved in patient care	
Steve	Prescott			Leeds Teaching Hospitals NHS Trust	Leeds, UK	Medical staff involved in patient care	
Hannah	Roberts			Leeds Teaching Hospitals NHS Trust	Leeds, UK	Research Nurse	
Angela	Morgan			Leeds Teaching Hospitals NHS Trust	Leeds, UK	Research Nurse	
Chris	Main			Leeds Teaching Hospitals NHS Trust	Leeds, UK	Research Nurse	
Elspeth	Bedford			Leeds Teaching Hospitals NHS Trust	Leeds, UK	Admin and secretarial support	
Lorraine	Wiseman			Leeds Teaching Hospitals NHS Trust	Leeds, UK	Research Nurse	
Bernice	Mpofu			Leeds Teaching Hospitals NHS Trust	Leeds, UK	Medical staff involved in patient care	
Claire	Daisey			Leeds Teaching Hospitals NHS Trust	Leeds, UK	Research Nurse	
Michelle	Donachie			Leeds Teaching Hospitals NHS Trust	Leeds, UK	Research Nurse	
Jon	Aning			North Bristol NHS Trust	Bristol, UK	Medical staff involved in patient care	
Lyndsey	Johnson			North Bristol NHS Trust	Bristol, UK	Research Nurse	
Carol	Brain			North Bristol NHS Trust	Bristol, UK	Medical staff involved in patient care	
Constance	Shiridzinomwa			North Bristol NHS Trust	Bristol, UK	Research Nurse	
Martin	Ebon			Lister NHS Hospital, Stevenage	Stevenage, UK	Medical staff involved in patient care	

Supplemental Online Content: Nonauthor Collaborators
*First name, last name, and suffix (if applicable) are required and will appear in PubMed.

***First Name and Middle Initial(s)**	***Last Name**	***Suffix (eg, Jr, III)**	Academic Degrees	Institution	Location (city, state/province, country)	Role or Contribution, eg, chair, principal investigator	Group (if more than 1 Group listed in the byline) and/or Subgroup (eg, Steering Committee)
Alexander	Hampson			Lister NHS Hospital, Stevenage	Stevenage, UK	Medical staff involved in patient care	
Roisin	Schimmel			Lister NHS Hospital, Stevenage	Stevenage, UK	Medical staff involved in patient care	
Scott	Horsley			Lister NHS Hospital, Stevenage	Stevenage, UK	Medical staff involved in patient care	
Sayyida	Nembhard			Lister NHS Hospital, Stevenage	Stevenage, UK	Medical staff involved in patient care	
Clare	Collins			Lister NHS Hospital, Stevenage	Stevenage, UK	Medical staff involved in patient care	
Jemma	Gilmore			Lister NHS Hospital, Stevenage	Stevenage, UK	Research Nurse	
Faith	Wilson			Lister NHS Hospital, Stevenage	Stevenage, UK	Medical staff involved in patient care	
Louise	Peacock			Lister NHS Hospital, Stevenage	Stevenage, UK	Medical staff involved in patient care	
Sheena	Lim			Lister NHS Hospital, Stevenage	Stevenage, UK	Research Nurse	
Rhosyll	Gabriel			Lister NHS Hospital, Stevenage	Stevenage, UK	Research Nurse	
Rachael	Sarpong			Surgical & Interventional Trials Unit	London, UK	UK Trials unit staff	
Melanie	Tan			Surgical & Interventional Trials Unit	London, UK	UK Trials unit staff	
