# Supplementary material for: Cost-Effectiveness of Robot-Assisted Radical Cystectomy vs Open Radical Cystectomy for Patients With Bladder Cancer
Source: JAMA Netw Open. 2023 Jun 30;6(6):e2317255. doi: 10.1001/jamanetworkopen.2023.17255 (PMC10314306; doi:10.1001/jamanetworkopen.2023.17255)
Supplement: Supplement 3. — Data Sharing Statement [file jamanetwopen-e2317255-s003.pdf]

Data Sharing Statement

Dixon. Cost-effectiveness of Robot-Assisted Radical Cystectomy vs Open Radical Cystectomy for Patients With Bladder Cancer. *JAMA Netw Open*. Published June 30, 2023. doi:10.1001/jamanetworkopen.2023.17255

Data
Data available: No

Additional Information
Explanation for why data not available: The underlying trial data are available for specified meta-analysis via the original article: Catto JWF, Khetrapal P, Ricciardi F, et al; iROC Study Team. Effect of robot-assisted radical cystectomy with intracorporeal urinary diversion vs open radical cystectomy on 90-day morbidity and mortality among patients with bladder cancer: a randomized clinical trial. JAMA. Published online May 15, 2022. doi:10.1001/jama.2022.7393
